# E3 Ubiquitin Ligase HRD1 Promotes Lung Tumorigenesis by Promoting Sirtuin 2 Ubiquitination and Degradation

**DOI:** 10.1128/MCB.00257-19

**Published:** 2020-03-16

**Authors:** Liu Liu, Le Yu, Cheng Zeng, Hua Long, Guangjie Duan, Guobing Yin, Xiaotian Dai, Zhenghong Lin

**Affiliations:** a School of Life Sciences, Chongqing University, Chongqing, People’s Republic of China; b Department of Pulmonology, Southwest Hospital, Third Military Medical University, Chongqing, People’s Republic of China; c Department of Breast, Thyroid, Pancreatic Surgery, The Second Affiliated Hospital of Chongqing Medical University, Chongqing, People’s Republic of China

**Keywords:** SIRT2, HRD1, ubiquitination, lung cancer, histone deacetylase, SIRT2

## Abstract

The NAD-dependent histone deacetylase sirtuin 2 (SIRT2) plays critical roles in mitosis and cell cycle progression and recently was shown to suppress tumor growth and to be downregulated in several types of cancers. However, the underlying mechanism of SIRT2 downregulation remains unknown. In this study, using bioinformatics, gene expression profiling, protein overexpression approaches, and cell migration assays, we showed that E3 ubiquitin ligase 3-hydroxy-3-methylglutaryl coenzyme A (HMG-CoA) reductase degradation 1 (HRD1) interacts with SIRT2 and promotes its ubiquitination and degradation. Furthermore, we found that HRD1 deficiency induces SIRT2 upregulation and inhibits the growth and tumor formation of lung cancer cells both *in vitro* and *in vivo*. Of note, we observed that SIRT2 expression is downregulated in human lung cancer and also negatively correlates with HRD1 expression in these cancers. Additionally, we found that patients with lung adenocarcinoma having lower HRD1 or higher SIRT2 expression levels tend to survive longer. On the basis of these results, we propose a mechanism of lung tumorigenesis that involves HRD1-mediated downregulation of SIRT2 and suggest that interventions targeting HRD1 activity could be a potential therapeutic strategy to treat patients with lung cancer.

## Abstract (precis)

The NAD-dependent histone deacetylase sirtuin 2 (SIRT2) plays critical roles in mitosis and cell cycle progression and recently was shown to suppress tumor growth and to be downregulated in several types of cancers. However, the underlying mechanism of SIRT2 downregulation remains unknown. In this study, using bioinformatics, gene expression profiling, protein overexpression approaches, and cell migration assays, we showed that E3 ubiquitin ligase 3-hydroxy-3-methylglutaryl coenzyme A (HMG-CoA) reductase degradation 1 (HRD1) interacts with SIRT2 and promotes its ubiquitination and degradation.

## INTRODUCTION

Lung cancer is the most commonly diagnosed cancer worldwide and also one of the three major causes of cancer-related death (1, 2). Approximately 57% of lung cancers are diagnosed at an advanced stage (3). The lack of reliable markers and therapeutic targets makes it difficult to diagnose and treat this aggressive disease. The identification of new targets with diagnostic and therapeutic value for lung cancer has become urgent. Sirtuin family members have been implicated in a variety of cellular functions, including gene silencing, DNA repair, cellular senescence, genomic stability, energy metabolism, and tumorigenesis (4–9). One member of the sirtuin family, SIRT2, plays crucial roles in neurodegeneration, mitosis, and cell cycle progression (10–15). SIRT2 plays important roles in the cell cycle by functioning as histone H4 lysine 16 (H4K16Ac) (10) and tubulin (11) deacetylases during mitosis. The checkpoint kinase BubR1 has also been shown to be a deacetylation target of SIRT2. What is more, the overexpression of SIRT2 in BubR1^H/H^ mice has been found to increase their life span (16). Cells with SIRT2 overexpression have shown significant cell cycle prolongation (17). In addition, SIRT2 has been shown to indirectly inhibit peroxisome proliferator-activated receptor gamma (PPARγ)-mediated adipocyte differentiation by reducing the amount of acetylation and phosphorylation of FOXO1 (18). A recent study suggested that SIRT2 may regulate the synthesis of fatty acids through the deacetylation of ACLY (19). SIRT2 plays an important role in tumorigenesis and tumor progression and is frequently downregulated in various cancers, including human breast, liver, glioma, renal, prostate, lung, uterine, and basal cell carcinomas (20–26). SIRT2 protein has also exhibited low expression in head and neck squamous cell carcinoma and esophageal adenocarcinoma (27, 28). These studies suggested that SIRT2 acts as a tumor suppressor (21) and could become a therapeutic target in cancer treatment (29). However, its function and how it is regulated in tumorigenesis remain unclear. Revealing the regulatory mechanism of SIRT2 in suppressing tumor formation would be of great significance in cancer therapy.

HRD1 (also called SYVN1), an E3 ubiquitin ligase that is involved in endoplasmic reticulum (ER)-associated degradation (ERAD), accepts ubiquitin specifically from the ER-associated UBC7 E2 ligase and transfers ubiquitin to substrates to promote their degradation (30, 31). The first identified substrate of HRD1 was 3-hydroxy-3-methylglutaryl coenzyme A (HMG-CoA) reductase (HMGCR), which is tightly regulated by metabolic feedback control (32). Later, it was reported that HRD1 also targets non-ERAD proteins to regulate various cellular processes (33–39) besides the ERAD component IRE1 (40). However, its involvement in lung tumorigenesis remains unknown.

In this study, we demonstrated that HRD1 can specifically interact with SIRT2 among the members of the mammalian SIR2 family (SIRT1 to -7). Interestingly, HRD1 is associated with and catalyzes SIRT2 ubiquitination and also promotes its degradation via ubiquitination. While SIRT2 has been identified as a tumor suppressor (21), these studies might help explain the novel mechanism by which HRD1 regulates tumorigenesis through SIRT2.

## RESULTS

### The protein level of SIRT2 was upregulated through HRD1 deficiency.

**FIG 1:**
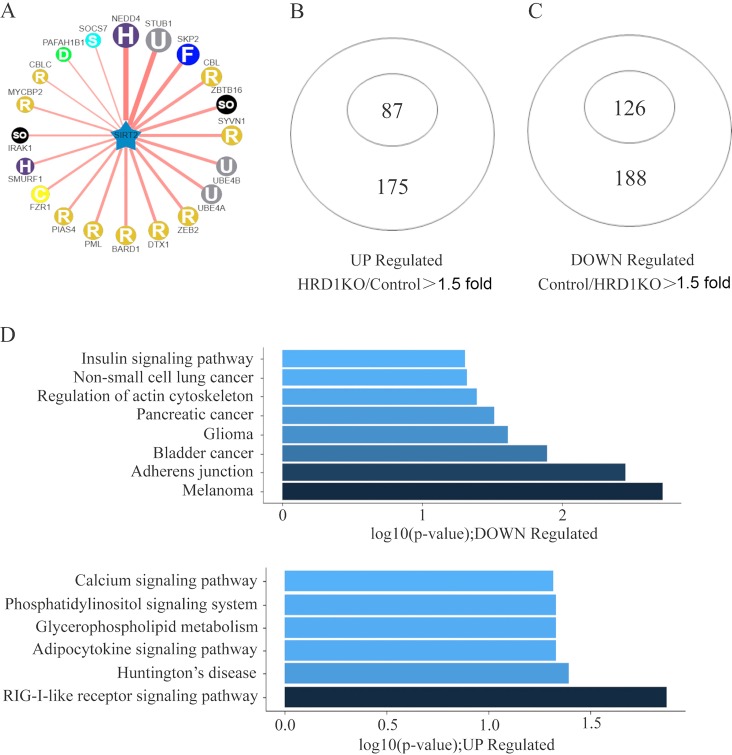
HRD1 deficiency upregulated the expression of SIRT2 as determined using mass spectrometry analysis. (A) The network view of the predicted E3 ligase of SIRT2 by UbiBrowser. The query substrate is located in the center of the canvas. The predicted E3 ligases surround the substrate. The node colors and characters reflect the E3 type. The edge width, the node size, and the edge shade are corrected with the confidence score. Node size, edge color depth, and edge width are proportional to the confidence score. H, HECT; U, UBOX; F, F-box; R, RING; C, CDC20; D, DWD; S, SOCS. (B and C) Proteomic analysis of genes differentially expressed between control and HRD1 knockout A549 cells. Diagrams depict 87 HRD1-repressed genes and 126 HRD1-activated genes. (D) Representative showing of HRD1-activated and -repressed genes by KEGG analysis.

The dysregulation of SIRT2 has been found to play a role in cancer progression and has also been suggested as a tumor suppressor (21, 41). However, the mechanism underlying its downregulation in tumor progression has been unclear. We used UbiBrowser software (http://ubibrowser.ncpsb.org/ubibrowser/home/index) (42) to predict the E3 ubiquitin ligase of SIRT2 and found that SYVN1 (HRD1) was a candidate (Fig. 1A). To test this possibility, we compared the gene expression patterns in the control and in HRD1 knockout A549 cells. A total of 213 genes encoding label-free proteins in the proteome were classified into two groups of genes with significantly different protein levels. The protein levels of the two gene groups increased >1.5-fold after HRD1 knockout. Figure 1B and Fig. S1A in the supplemental material show 87 genes upregulated upon HRD1 knockout, and Fig. 1C and Fig. S1B depict 126 genes downregulated upon HRD1 knockout. Interestingly, SIRT2 was detected in the upregulated genes, suggesting that HRD1 might be the E3 ubiquitin ligase of SIRT2.

### HRD1 interacts with SIRT2.

**FIG 2:**
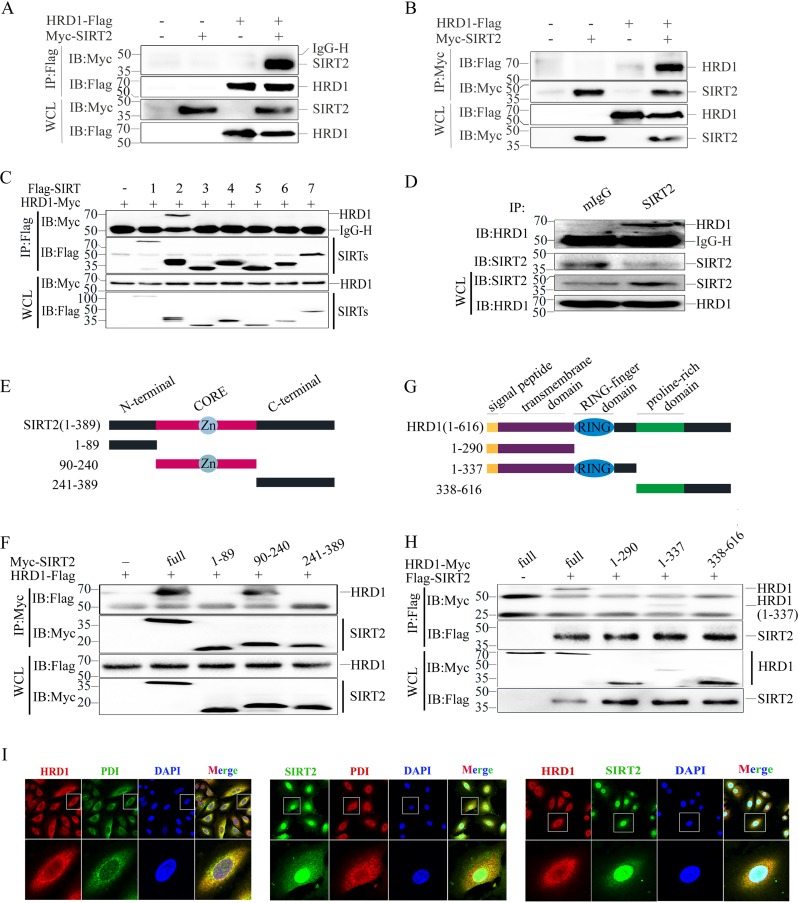
SIRT2 interacts with HRD1. (A) The Myc-SIRT2 expression plasmid was cotransfected with or without HRD1-Flag into HEK293T cells. The SIRT2 protein was immunoprecipitated (IP) with the anti-Flag antibody, and the expression of SIRT2 and HRD1 in the whole-cell lysate (WCL) was confirmed by immunoblotting (IB) with antibodies against Myc and Flag. (B) The HRD1-Myc expression plasmid was cotransfected with or without Flag-SIRT2 into HEK293T cells. The HRD1 protein was immunoprecipitated with anti-Flag antibody, and the expression of SIRT2 and HRD1 in the WCL was confirmed by IB with antibodies against Myc and Flag. (C) HEK293T cells were cotransfected with the HRD1-Myc expression plasmid and Flag-SIRT1 to Flag-SIRT7. The HRD1 protein was immunoprecipitated with the anti-Flag antibody. The expression of HRD1 and SIRTs in the WCL was confirmed by IB with antibodies against Myc and Flag. (D) The endogenous interaction of HRD1 and SIRT2 was evaluated in A549 cells. A normal mouse IgG (mIgG) was used as a control. (E) Schematic representation of SIRT2 and its mutants, indicating that SIRT2 contains a CORE domain. (F) Schematic representation of HRD1 and its mutants, showing that HRD1 protein contains an N-terminal signal peptide, transmembrane domain, zinc finger domain, and C-terminal proline-rich domain. (G) The interactions of SIRT2 and its mutants with HRD1 protein were evaluated. HRD1-Flag plasmids were cotransfected with SIRT2 or each of its mutants, and their interactions were analyzed as for panel A. (H) The interactions of SIRT2 with HRD1 and its mutants were evaluated. Flag-SIRT2 plasmids were cotransfected with HRD1 or each of its mutants into HEK293T cells, and their interactions were analyzed as for panel A. For panels A to D, F, and H, numbers on the left are molecular masses, in kilodaltons. (I) Colocalization of HRD1 and PDI, SIRT2 and PDI1, and HRD1 and SIRT2 in HeLa cells. The cellular localization of HRD1, SIRT2, and PDI was examined by immunofluorescence staining with corresponding antibodies. 4,6-Diamidino-2-phenylindole (DAPI) was used to stain the DNA.

To test the possibility of HRD1 as the E3 ubiquitin ligase candidate of SIRT2 (Fig. 1), we first investigated the interaction between HRD1 and SIRT2. HRD1 was shown to pull down SIRT2, and conversely, SIRT2 was shown to pull down HRD1 (Fig. 2A and B). HRD1 was able to specifically interact with SIRT2 among the seven members of the gene family (SIRT1 to -7) (Fig. 2C). Next, we demonstrated that endogenous HRD1 and SIRT2 proteins also interact with one another in A549 cells (Fig. 2D). Furthermore, we mapped the regions of SIRT2 protein involved in the interaction with HRD1 by constructing a series of SIRT2 truncated mutants (Fig. 2E). These mutants were cotransfected with HRD1 into HEK293T cells, and coimmunoprecipitation showed that the full-length SIRT2 and the truncated mutants (residues 90 to 240) interacted with HRD1 (Fig. 2F). HRD1 contains a signal peptide at its N terminus, a transmembrane domain, a RING finger domain that is necessary for HRD1 ubiquitin ligase activity, and a C-terminal, proline-rich domain (43) (Fig. 2G). We generated a series of HRD1 deletion mutants and transfected HEK293T cells with SIRT2 to identify the regions of HRD1 responsible for the SIRT2 interaction. Full-length HRD1 (residues 1 to 616) and the N terminus of HRD1 containing the RING-finger domain (residues 1 to 337) interacted with SIRT2 (Fig. 2H). Finally, we confirmed that both endogenous HRD1 and SIRT2 colocalized in the endoplasmic reticulum (ER) of the cells via immunofluorescence staining (Fig. 2I). Taken together, these results suggested that HRD1 interacts with SIRT2 and that the interaction might be mediated by the RING finger domain and the CORE domain of HRD1 and SIRT2, respectively.

### HRD1 promotes SIRT2 ubiquitination.

**FIG 3:**
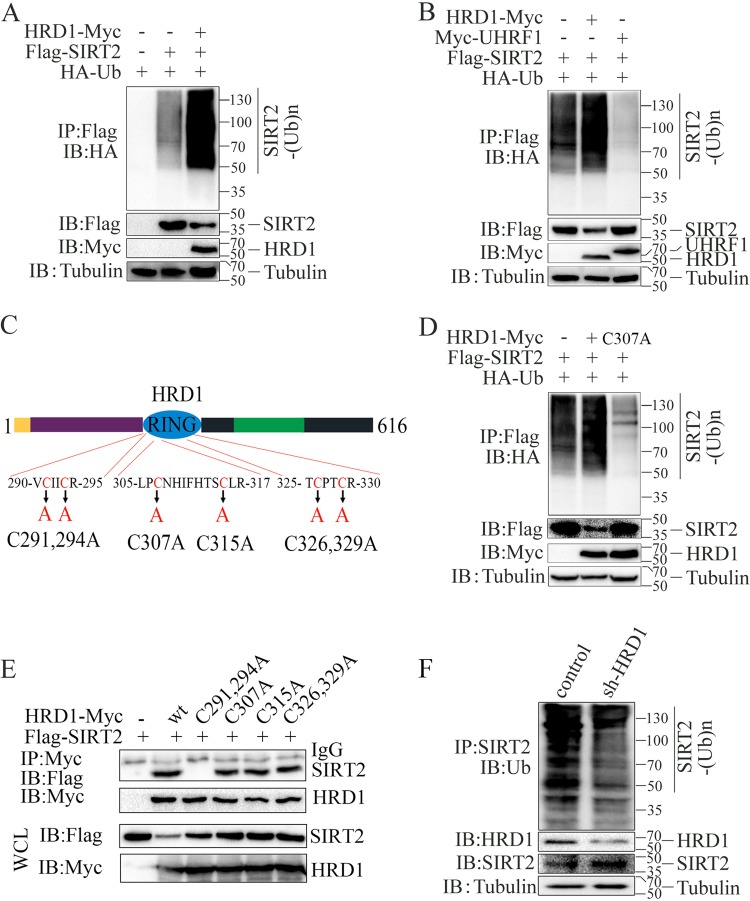
HRD1 promotes SIRT2 ubiquitination. (A) HA-ubiquitin, Flag-SIRT2, and HRD1-Myc plasmids were cotransfected into HEK293T cells. SIRT2 ubiquitination was detected by immunoprecipitation of SIRT2 with the anti-Flag antibody and Western blotting with anti-HA antibody. The protein expression levels of HA-ubiquitin, SIRT2, and HRD1 in the whole-cell lysates were confirmed. (B) HA-ubiquitin and Flag-SIRT2 expression plasmids were cotransfected with HRD1-Myc or Myc-UHRF1 into HEK293T cells. SIRT2 ubiquitination was analyzed as for panel A. (C) Schematic representation of HRD1 and its point mutants. The conserved cysteine (C) residues in the RING domain were replaced with alanine (A). (D) HA-ubiquitin and Flag-SIRT2 expression plasmids were cotransfected into HEK293T cells with HRD1-Myc or with HRD1/CA mutants. The effects of HRD1 and its mutants on SIRT2 ubiquitination were analyzed as for panel A. (E) The interactions of SIRT2 with wild-type (wt) HRD1 or its mutants were analyzed as indicated for panel C. (F) HA-ubiquitin expression plasmids were transfected into the control and the stable knockdown HRD1 A549 cancer cells. SIRT2 ubiquitination was detected via immunoprecipitation of SIRT2 with anti-SIRT2 antibody and Western blotting with anti-UB antibody. The protein expression levels of SIRT2 and HRD1 in the whole-cell lysates were confirmed.

As an E3 ligase, HRD1 could also use the ubiquitin-proteasome system to degrade targeted proteins (31). Therefore, to evaluate whether HRD1 could promote SIRT2 ubiquitination, we transfected HRD1, ubiquitin (Ub), and SIRT2 into HEK293T cells and then analyzed their expressions. Polyubiquitinated SIRT2 proteins were detected using an antihemagglutinin (anti-HA) antibody (Fig. 3A). Additionally, HRD1-mediated SIRT2 ubiquitination was specific, in contrast to the ubiquitination by UHRF1, a member of a subfamily of RING finger-type E3 ubiquitin ligases (Fig. 3B). Furthermore, SIRT2 ubiquitination depended on E3 ubiquitin ligase catalytic activity, but compared with the wild-type HRD1, the catalytically inactive E3 ubiquitin ligase HRD1/CA mutant (Fig. 3C) did not promote SIRT2 ubiquitination (Fig. 3D). Interestingly, we found that the C291, 294A mutant of HRD1 did not interact with SIRT2 (Fig. 3E). Moreover, we demonstrated that the knockdown of endogenous HRD1 via small hairpin RNA (shRNA) decreased the ubiquitination of endogenous SIRT2 in A549 cells (Fig. 3F). Collectively, these results suggested that HRD1 is a specific E3 ubiquitin ligase for SIRT2 and that HRD1 positively regulates SIRT2 protein ubiquitination.

### HRD1 negatively regulates SIRT2 protein stability.

**FIG 4:**
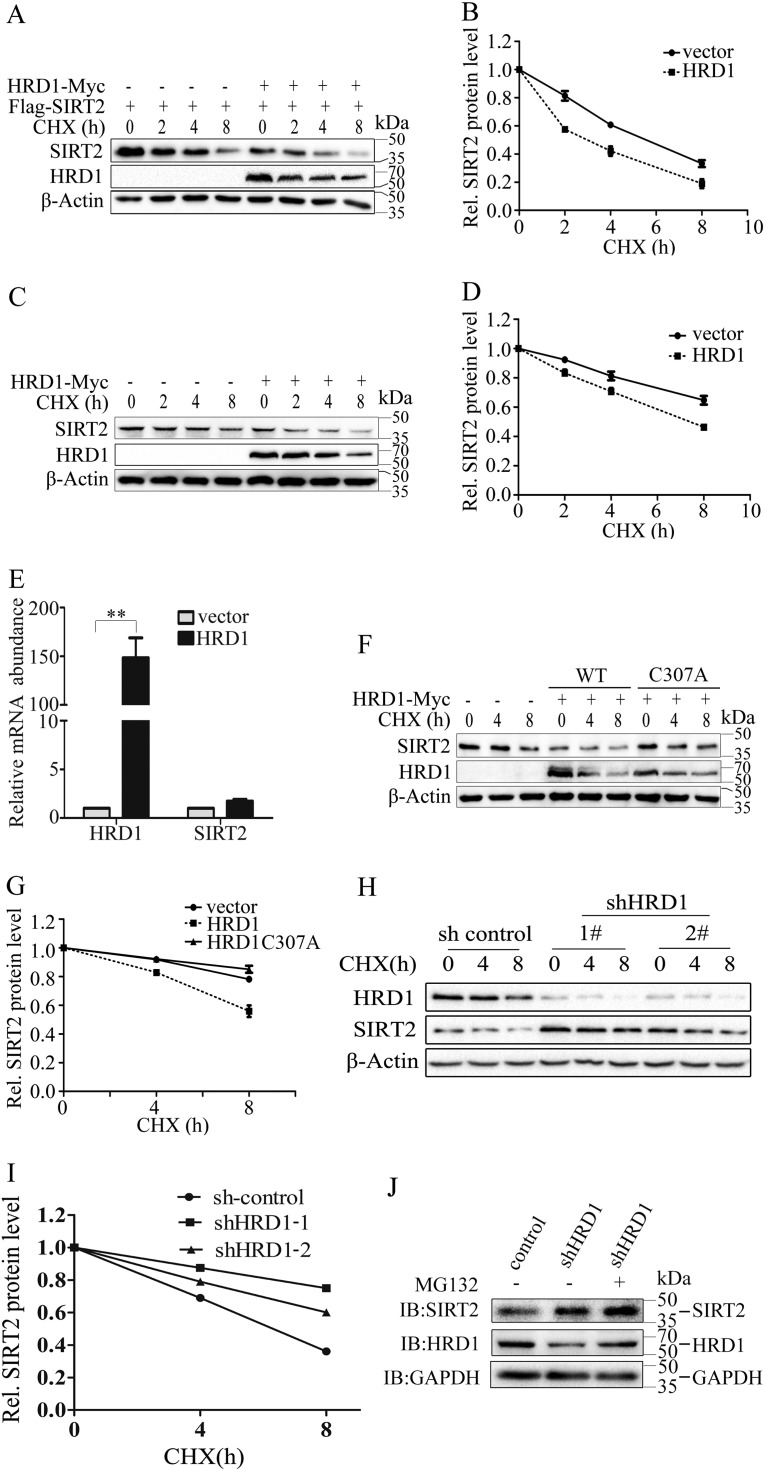
HRD1 negatively regulates SIRT2 protein stability. (A and B) HRD1 expression plasmids or empty vectors were cotransfected with Flag-SIRT2 into HEK293T cells. The transfected cells were treated with cycloheximide (CHX) for different times. The protein levels in the treated cells were determined by Western blotting using anti-Flag (A, top) and anti-Myc (A, middle) antibodies (Abs). β-Actin was used as a loading control (A, bottom). The band intensities of SIRT2 proteins were quantified, and their relative levels are shown in panel B. (C and D) HRD1 expression plasmids or empty vectors were transfected into HEK293T cells. The transfected cells were treated with CHX for different times. The protein levels in the treated cells were determined through Western blotting using anti-SIRT2 (C, top) and anti-Myc (C, middle) Abs. β-Actin was used as a loading control (C, bottom). The band intensities of SIRT2 proteins were quantified, and their relative levels are shown in panel D. (E) A fraction of cells from panel A was prepared in parallel for total RNA extraction. The mRNA levels of both SIRT2 and HRD1 were determined by real-time PCR. Their relative levels are indicated. The error bar represents the SEMs from triplicate experiments. **, *P* < 0.05, two-tailed Student’s *t* test. (F and G) HRD1 or HRD1/CA mutant plasmids were transfected into HEK293T cells. SIRT2 protein stabilities in the transiently transfected HEK293T cells were examined as described for panels A and B. (H and I) A549 cells stably expressing control shRNA or HRD1-specific shRNA (shHRD1-1 and shHRD1-2) were treated with CHX for the indicated time. SIRT2 protein stabilities were analyzed as described for panel A (H, top). The expression levels of HRD1 (H, middle) were confirmed through Western blotting using β-actin as a loading control (H, bottom). The band intensities of SIRT2 proteins were quantified, and their relative levels are shown in panel I. (J) A549 cells stably expressing control or HRD1 knockdown plasmids were treated with the proteasome inhibitor MG132. The protein levels of SIRT2 (top) and HRD1 (middle) were determined by Western blotting with GAPDH as a loading control (bottom).

Ubiquitination often promotes protein degradation. Our discovery that HRD1 ubiquitinated SIRT2 implies that HRD1 might regulate SIRT2 protein stability. To determine whether HRD1 could promote ubiquitination-mediated SIRT2 degradation, we transfected HRD1 and SIRT2 plasmids into HEK293T cells and measured the SIRT2 protein level. As expected, HRD1 significantly shortened the half-life of the SIRT2 protein (Fig. 4A and B). HRD1 overexpression downregulated endogenous SIRT2 protein levels (Fig. 4C and D). Moreover, HRD1 acted as a regulator of SIRT2 protein stability rather than the expression of mRNA (Fig. 4E). The catalytically inactive HRD1/CA mutant failed to degrade SIRT2 (Fig. 4F and G). To further investigate whether endogenous HRD1 could promote SIRT2 protein degradation, we knocked down HRD1 using shRNA in A549 cells and measured the SIRT2 protein half-lives with the cycloheximide (CHX) chase assay. The SIRT2 protein half-life was decreased when HRD1 was depleted (Fig. 4H and I). Moreover, we found that MG132, a proteasome-specific inhibitor, protected SIRT2 from degradation (Fig. 4J), which suggested that SIRT2 protein degradation also occurs through the proteasome pathway. Taken together, these results suggested that HRD1 promotes SIRT2 ubiquitin-mediated degradation through the proteasome pathway.

### HRD1 promotes cell proliferation and tumorigenesis in lung cancer.

**FIG 5:**
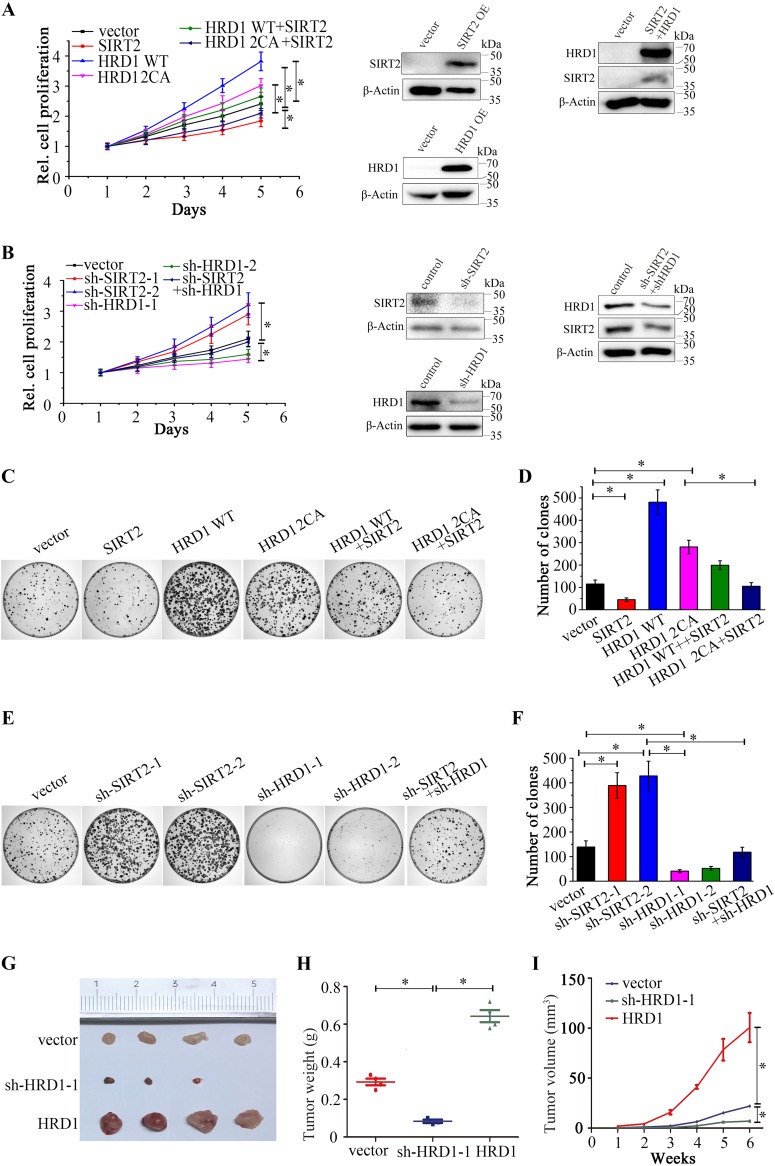
HRD1 knockdown suppresses lung cancer cell proliferation and tumorigenesis. (A and B) The cell proliferation of A549 cancer cells stably expressing the indicated plasmids or combination of plasmids was determined by the 3-(4,5-dimethylthiazol-2-yl)-5-(3-carboxymethoxyphenyl)-2-(4-sulfophenyl)-2H-tetrazolium (MTS) assay. The error bars represent the SEMs from triplicate experiments. HRD1 2CA mutant C291, 294A was used. The protein levels of SIRT2, HRD1, or both were determined by Western blotting using β-actin as a loading control in A549 cells stably expressing control vector or indicated overexpression or knockdown plasmids. (C to F) A clonogenic assay was performed to measure the colony formation capacity of A549 cancer cells stably expressing the indicated plasmids (C) or stably expressing the indicated knockdown plasmids (E). The quantitation of colony number is shown in panels D and F. Error bars represent the SD from triplicate experiments. *, *P* < 0.05. (G to I) A549 cells stably expressing control, HRD1 knockdown, and HRD1 plasmids were injected subcutaneously into nude mice. Six weeks after injection, tumors were isolated and photographed (G). The tumor sizes were measured and are depicted as tumor weight (H) and tumor volume (I).

SIRT2 has been identified as a tumor suppressor (21). Therefore, our hypothesis was that HRD1 can promote cell proliferation by regulating SIRT2 protein levels. To test this hypothesis, we assessed the biological role of HRD1 in lung cancer by investigating the effects of HRD1 overexpression and HRD1 knockdown on the viability and colony formation of A549 and H446 cancer cells. As expected, HRD1 overexpression increased the tumor cell growth of both A549 and H446 cancer cells (Fig. 5A and Fig. S2A). Notably, an SIRT2 interaction deficiency mutant of HRD1 had a much weaker effect on cell proliferation than wild-type HRD1. HRD1 knockdown via shRNA appeared to inhibit the proliferation of A549 and H446 cancer cells (Fig. 5B and Fig. S2B), while SIRT2 overexpression or knockdown led to the reverse result (Fig. 5A and B and Fig. S2A and B). Colony formation assay further confirmed that the stable overexpression of HRD1 in either A549 or H446 cancer cells significantly enhanced colony formation (Fig. 5C and D and Fig. S2C and D), and the stable knockdown of HRD1 resulted in a dramatic decrease in colony numbers (Fig. 5E and F and Fig. S2E and F). The enhancement of lung cancer cell proliferation and colony formation was partially abrogated by the overexpression of either HRD1 or SIRT2. The simultaneous loss of HRD1 and SIRT2 cells partially restored cell proliferation and colony formation. This finding suggested that HRD1 enhances lung cancer cell growth. We further examined whether HRD1 affects tumorigenesis *in vivo*; results are depicted in Fig. 5G to I. Using a mouse xenograft model, we found a dramatically smaller tumor size in HRD1-depleted cells and larger tumor size in HRD1-overexpressing cells. Collectively, these findings suggested that the depletion of HRD1 inhibits tumor growth.

### HRD1 promotes lung cancer cell migration and invasion.

**FIG 6:**
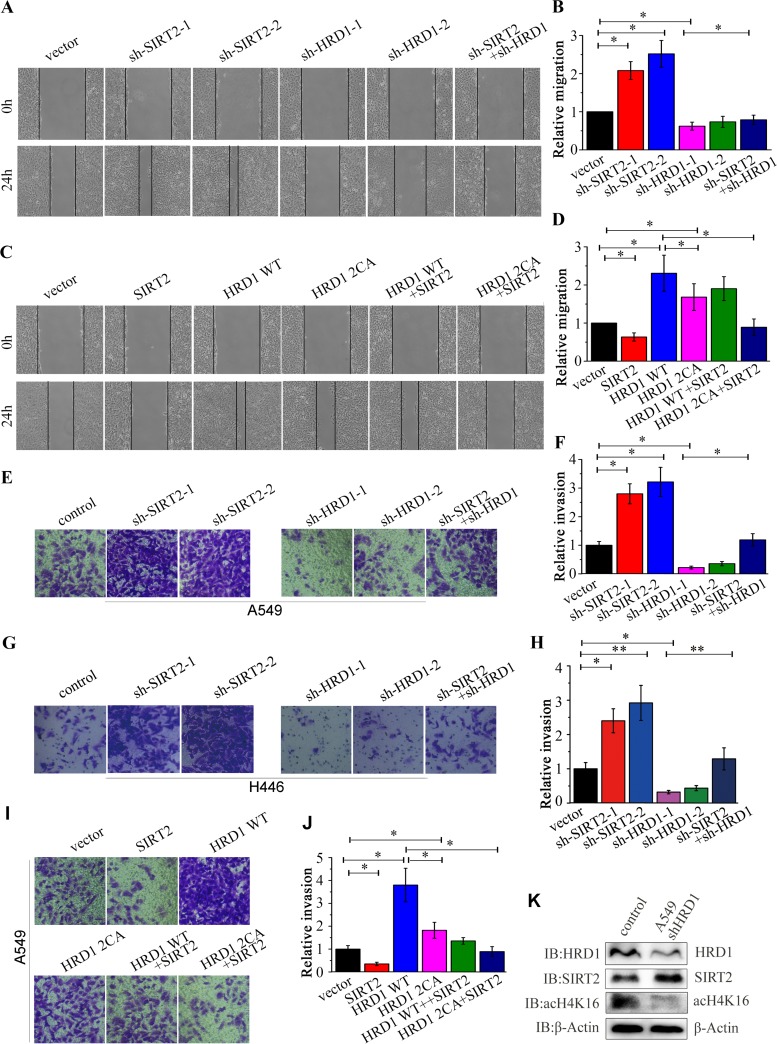
HRD1 knockdown inhibits cell migration and invasion in lung cancer cells. (A to D) Wound-healing assays were performed to measure the migration of A549 cells stably expressing the indicated knockdown or overexpression plasmids or a combination of plasmids. Cells were monitored within 24 h to evaluate the rate of migration into the scratched area. The wound edges are indicated by black lines in panels A and C. The relative distances of migration are shown in panels B and D. The results represent the means ± SEMs from triplicate experiments. *, *P* < 0.05; **, *P* < 0.01. (E to J) The effects of stable knockdown (E to H) or overexpression (I and J) of HRD1 or SIRT2 on cell invasion were measured in A549 cells (E, F, I, and J) or H446 cells (G and H) by Matrigel Transwell assays. The quantitative results are shown. The results represent the means and SD from triplicate experiments. *, *P* < 0.05; **, *P* < 0.01. (K) The protein levels of HRD1, SIRT2, ac-H4K16 were determined by Western blotting using β-actin as a loading control in A549 cells stably expressing control or HRD1 knockdown plasmids.

Next, we explored the roles of the HRD1-SIRT2 interaction in lung cancer metastasis by evaluating cell migration and invasion. The wound-healing assay showed that the knockdown of SIRT2 significantly increased cell migration (Fig. 6A and B and Fig. S2I and J) compared to that in the control cells, whereas the overexpression of SIRT2 showed the reverse effect (Fig. 6C and D and Fig. S2G and H). The knockdown of HRD1 decreased cell migration (Fig. 6A and B and Fig. S2I and J), whereas the overexpression of HRD1 reversed the phenotype (Fig. 6C and D and Fig. S2G and H). Interestingly, the SIRT2 interaction deficiency mutant of HRD1 had a much weaker effect on colony formation and cell migration than did wild-type HRD1. In addition, the knockdown of SIRT2 in HRD1-depleted cells and the introduction of SIRT2 into HRD1-overexpressing cells both partially restored cell migration (Fig. 6A to D). The Matrigel Transwell invasion assays consistently showed similar results regarding the role of HRD1 and SIRT2 in the tumor invasion of A549 (Fig. 6E, F, I, and J) and H446 (Fig. 6G and H) cells. Taken together, these data revealed that HRD1 promotes lung cancer cell migration and invasion. To investigate whether the biological activity of SIRT2 could be regulated by HRD1, we assessed the acetylation level of H4K16 and found that it was depleted when HRD1 was downregulated, which suggested that HRD1 may regulate SIRT2 activity (Fig. 6K).

### SIRT2 and HRD1 expressions are inversely correlated in lung cancer samples.

**FIG 7:**
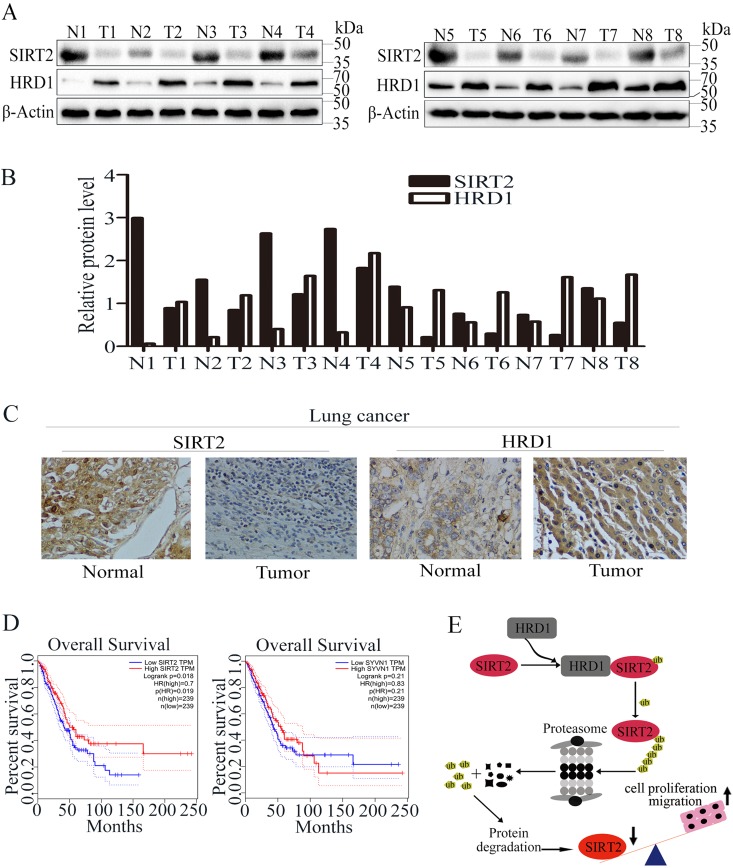
HRD1 and SIRT2 expression in lung cancer. (A) The protein levels of HRD1 and SIRT2 were measured in lung cancer tissue specimens (T; *n* = 8) and matched with adjacent normal tissues (N; *n* = 8) through Western blotting. (B) The relative HRD1 and SIRT2 protein levels in normal and cancer tissues were quantified. (C) Representative images showing the immunohistochemical staining of HRD1 and SIRT2 in normal and lung cancer tissues. (D) Analysis of the GEPIA data indicates a significant positive correlation of SIRT2 expression and a negative correlation of HRD1 expression between their expression and the overall survival of lung adenocarcinoma patients. GEPIA uses the log rank test, also known as the Mantel-Cox test, for the hypothesis test. The Cox proportional hazard ratio and the 95% confidence interval information are also included in the survival plot. (E) Schematic model of SIRT2 regulation by HRD1. Upregulation of HRD1 in lung cancer patients leads to downregulation of SIRT2, thus resulting in enhanced cell proliferation and lung tumorigenesis.

Studies have shown that SIRT2 is remarkably downregulated in human breast cancers and hepatocellular carcinoma (HCC) samples. To investigate whether SIRT2 could have a similar expression pattern in lung cancer cells, we examined the expression levels of HRD1 and SIRT2 in lung cancer tissues (*n* = 8) and matched adjacent normal lung tissues (*n* = 8) using Western blotting (Fig. 7A). As Fig. 7B shows, the relative HRD1 and SIRT2 protein levels were obviously negatively correlated. The relationship was further examined via the immunohistochemical staining of lung cancer tissues and normal tissues. As Fig. 7C shows, the expression level of HRD1 significantly increased, whereas SIRT2 was downregulated in lung cancer tissues as opposed to matched normal lung tissues. We then analyzed the correlation between the overall survival of lung adenocarcinoma patients and HRD1 or SIRT2 expression by using Gene Expression Profiling Interactive Analysis (GEPIA) transcriptome sequencing (RNA-Seq) data sets (44). Higher expression of HRD1 in lung adenocarcinoma patients predicted worse survival rates for the patients (Fig. 7D). In contrast, SIRT2 expression was positively correlated with the overall survival of lung adenocarcinoma patients (Fig. 7D). However, precautions had to be taken when drug design based on this result was applied because the cancer diagnosis and individual stage could vary in these patients.

Based on our results, we concluded that SIRT2 is ubiquitinated by HRD1 and its protein level is negatively regulated and correlated with HRD1 expression. Upregulation of HRD1 in lung cancer patients leads to decreased SIRT2 expression and enhanced cell proliferation and tumorigenesis, and HRD1 and SIRT2 expression levels may serve as prognostic markers for lung cancer (Fig. 7E).

## DISCUSSION

Ample evidence indicates that the incidence of malignant tumors increases with age. SIRT2 is the major cytoplasmic sirtuin associated with age-related diseases. Among animals and humans, cancer is one of the major age-related diseases. Therefore, in the past few years, the potential direct role of sirtuins as longevity factors in tumorigenesis has been proposed as a very interesting hypothesis that warrants testing. As members of the sirtuin family, SIRT1 AND SIRT3 have been shown to possess a tumor suppressor function (45–50). The data regarding the role of SIRT2 in tumorigenesis have been somewhat conflicting. Some studies have indicated that SIRT2 might promote tumor formation through the deacetylation and inhibition of p53 (51, 52), whereas other reports have indicated that the SIRT2 expression level is downregulated in human gliomas. *In vitro* colony formation ability was even shown to be reduced when SIRT2 was ectopically expressed in glioma cell lines (20). In addition, it has been suggested that the absence of SIRT2 promotes genomic instability, a recognized early event in the development of cancer (7, 53–55). More importantly, one study also showed that Sirt2^−/−^ mice formed tumors in multiple tissues and that the incidence of the tumors increased slowly with age (21). Previous studies also showed that SIRT2 was significantly downregulated in non-small cell lung cancer (23, 25, 56). Our study showed that SIRT2 expression was downregulated in lung cancer and that this change was accompanied by HRD1 upregulation. This implied that HRD1 might promote tumor cell growth by promoting the ubiquitination and degradation of SIRT2 and that SIRT2 functions as a tumor suppressor.

In this study, we identified HRD1 as an SIRT2-interacting protein by coimmunoprecipitation and Western blotting. Additionally, the ubiquitination and degradation results revealed that SIRT2 is a direct substrate of HRD1. Furthermore, we demonstrated that HRD1 deficiency decelerates lung cancer cell proliferation and tumor formation and that SIRT2 knockdown restores the cell proliferation phenotype in HRD1 knockdown cells. In addition, we demonstrated that HRD1 promotes lung cancer cell metastasis and invasion by downregulating SIRT2 expression. Taken together, these results suggested that HRD1 is involved in regulating lung cancer tumorigenesis and metastasis through SIRT2 (Fig. 7E).

SIRT2 was reported to decrease in human gliomas, and colony formation ability was inhibited by the overexpression of SIRT2 in glioma cell lines *in vitro* (20). A recent study of genomic data also showed that the expression of SIRT2 was lower in human breast cancer and HCC samples than in normal human tissue samples (21). Furthermore, SIRT2 mRNA expression was reduced in anaplastic oligodendroglioma, glioblastoma, clear cell renal carcinoma, and prostate carcinoma (57). In our study, we showed that SIRT2 expression was positively correlated with the overall survival of lung adenocarcinoma patients but negatively correlated with HRD1 expression. The poor patient survival rate was associated with low SIRT2 levels (23). Other studies have also shown that the combination of SIRT1 and SIRT2 is a better predictive model of recurrence-free survival (RFS) in non-small cell lung cancer (NSCLC) to stratify patients (58).

In summary, our results revealed that HRD1 is an E3 ligase for SIRT2 that mediates ubiquitination and degradation. HRD1 stimulated cell growth, tumorigenesis, and migration through SIRT2. Our data, combined with previous results (25, 59–61) indicating that HRD1 mediates SIRT2, suggested that targeting the regulation of SIRT2 by repressing HRD1 expression may be an important method for NSCLC treatment.

## MATERIALS AND METHODS

### Ethics statement.

All patients who provided tissue samples signed forms for informed consent. We got approval from the institutional ethics body, and the approval number is 2016018. For Fig. 7A to C, a total of 8 pairs of tissue samples were collected based on the clinical diagnosis of pathological puncture biopsy specimen. For Fig. 7D, survival analysis of 478 patients was performed based on the GEPIA and the Cancer Genome Atlas (TCGA) database.

### Plasmids and antibodies.

HRD1 and SIRT2, along with their mutants, were cloned into pCMV-Flag 5a or pCMV-Myc (Addgene, USA). The lentiviral vector was purchased from Addgene, and packaging was performed in HEK293T cells. Nonspecific shRNA was used as a control. The sequences were as follows: for shCtrl, 5′-GATCCGTTCTCCGAACGTGTCACGTAATTCAAGAGATTACGTGACACGTTCGGAGAATTTTTTC-3′ (forward) and 5′-AATTGAAAAAATTCTCCGAACGTGTCACGTAATCTCTTGAATTACGTGACACGTTCGGAGAACG-3′ (reverse); for shHRD1-1, 5ʹ-CCGGACTGCAGTGCCGAACAGTATTCTCGAGAATACTGTTCGGCACTGCAGTTTTTTG-3ʹ (forward) and 5ʹ-AATTCAAAAAACTGCAGTGCCGAACAGTATTCTCGAGAATACTGTTCGGCACTGCAGT-3ʹ (reverse); for shHRD1-2, 5′-CCGGTGATGGGCAAGGTGTTCTTTGCTCGAGCAAAGAACACCTTGCCCATCATTTTTG-3′ (forward) and 5′-AATTCAAAAATGATGGGCAAGGTGTTCTTTGCTCGAGCAAAGAACACCTTGCCCATCA-3′ (reverse); for shSIRT2-1, 5ʹ-CCGGCCTGCTCATCAACAAGGAGAACTCGAGTTCTCCTTGTTGATGAGCAGGTTTTTG-3ʹ (forward) and 5ʹ-AATTCAAAAACCTGCTCATCAACAAGGAGAACTCGAGTTCTCCTTGTTGATGAGAGG-3ʹ (reverse); and for shSIRT2-2, 5′-CCGGGCCAACCATCTGTCACTACTTCTCGAGAAGTAGTGACAGATGGTTGGCTTTTTG-3′ (forward) and 5′-AATTCAAAAAGCCAACCATCTGTCACTACTTCTCGAGAAGTAGTGACAGATGGTTGGC-3′ (reverse). The following antibodies were used: anti-mouse SIRT2 (Zenbio, Chengdu, China), anti-rabbit actin (Sigma-Aldrich), anti-rabbit HRD1 (Sigma-Aldrich), anti-mouse Flag (Sigma-Aldrich), anti-mouse Myc (9E10; Santa Cruz Biotechnology), anti-rabbit ubiquitin (Ub) (Millipore), anti-rabbit HA (Santa Cruz Biotechnology), anti-rabbit glyceraldehyde-3-phosphate dehydrogenase (GAPDH) (Beyotime, Shanghai, China), anti-rabbit histone H4 (acetyl K16; Zenbio, Chengdu, China), anti-rabbit HIST4H4 (Sangon, Shanghai, China), and anti-mouse antitubulin (Sigma-Aldrich).

### Cell culture and transfection.

Cells from the A549 human lung cancer cell line were gifted by Zhong Luo (School of Life Sciences, Chongqing University). Cells from the HEK293T cell line (human embryonic kidney cells) and the human lung cancer cell line H446 were gifted by Li Zhong (Chongqing University). HEK293T cells were cultured in Dulbecco modified Eagle medium (DMEM; high glucose; Procell Life Science & Technology Co. Ltd., Wuhan, China) containing 10% fetal bovine serum (FBS) and 1% penicillin-streptomycin (P-S). Cells from the A549 and H446 human lung cancer cell lines were maintained in RPMI 1640 (Procell Life Science & Technology Co. Ltd.) containing 10% FBS and 1% P-S. All cells were maintained according to the supplier’s instructions and transfected with the desired plasmids using Lipofectamine 2000 (Invitrogen; 11668-019). When necessary, empty vectors were used to bring the total amount of DNA to equal amounts for each transfection group.

### Western blot analysis.

Total proteins were extracted and separated using sodium dodecyl sulfate-polyacrylamide gel electrophoresis (SDS-PAGE) and then transferred onto polyvinylidene difluoride (PVDF) membranes and immunoblotted with the desired antibodies.

### Ubiquitination analysis.

Cells were lysed with a high concentration of SDS lysis buffer, and immunoprecipitation with appropriate antibodies was conducted as described previously.

### Cycloheximide chase analysis.

Cells were transfected with the desired plasmids and grown for 24 h. CHX (100 mg/ml) was used to inhibit protein synthesis. Equal numbers of cells were collected at the desired time points. The SIRT2 levels in the samples were determined via immunoblotting as described above.

### RT-PCR.

Total RNA was extracted from HEK293T cells with TRIzol reagent according to the manufacturer’s protocol. Quantitative reverse transcription-PCR (RT-PCR) was performed using SYBR green quantitative PCR (qPCR) master mix. The β-actin gene was used as a reference for sample normalization. A standard amplification protocol was used according to the manufacturer’s instructions. The primers were as follows: HRD1 forward, 5ʹ-AACCCCTGGGACAACAAGG-3ʹ; HRD1 reverse, 5ʹ-GCGAGACATGATGGCATCTG-3ʹ; SIRT2 forward, 5ʹ-GAGGTGGCATGGATTTTGAC-3ʹ; and SIRT2 reverse, 5ʹ-AGATGGTAGTGCTGGGGTTG-3ʹ. β-Actin was used as a control using the following primers: forward, 5ʹ-CATGTACGTTGCTATCCAGGC-3ʹ, and reverse, 5ʹ-CTCCTTAATGTCACGCACGAT-3ʹ. Gene expression levels were analyzed using the threshold cycle (Δ*C_T_*) method.

### *In vivo* tumorigenesis.

Six-week-old nude mice were inoculated subcutaneously in the right hind flank with 5 × 10^6^ cells per 100 μl suspended in dilute Matrigel 1:1 in ice-cold phosphate-buffered saline (PBS). Tumor development was monitored over a period of 6 weeks before the mice were euthanized for further analysis. Tumor volume (in cubic millimeters) was measured with calipers and calculated as (*W*^2^ × *L*)/2, where *W* is the width and *L* is the length. All procedures involving animals were performed in accordance with the institutional animal welfare guidelines of Chongqing University.

### Cell apoptosis analysis.

Apoptosis was evaluated using the fluorescein isothiocyaate (FITC)-annexin V/dead cell apoptosis kit with FITC-annexin V and propidium iodide (PI) (Life Technologies, Carlsbad, CA), and the results were analyzed via flow cytometry.

### Proteomic analysis by MS.

The label-free tryptic peptides were dissolved in 20 μl 0.5% trifluoroacetic acid (TFA). The mass spectroscopy (MS) analysis was performed using a LTQ-XL ion trap mass spectrometer coupled online to an ultraperformance liquid chromatography (UPLC) instrument. The differentially expressed proteins were categorized as previously described (62).

### Statistical analysis.

The data are presented as the means and standard deviations (SD) from at least three independent experiments. Data were analyzed with Prism 5.0 (GraphPad Software, San Diego, CA). A *P* value of <0.05 was considered statistically significant.

## Supplementary Material

Supplemental file 1

Supplemental file 2

Supplemental file 3
