# Supplementary material for: E3 Ubiquitin Ligase HRD1 Promotes Lung Tumorigenesis by Promoting Sirtuin 2 Ubiquitination and Degradation
Source: Mol Cell Biol. 2020 Mar 16;40(7):e00257-19. doi: 10.1128/MCB.00257-19 (PMC7076256; doi:10.1128/MCB.00257-19)
Supplement: Supplemental file 1 [file MCB.00257-19-s0001.pdf]

The E3 ubiquitin ligase HRD1 promotes lung tumorigenesis by promoting sirtuin 2 ubiquitination and degradation

Figure S1 Proteomic analysis of genes differentially expressed between control and HRD1 knockout A549 cells. (**A&B**) 87 HRD1-repressed genes were marked with red color and 126 HRD1-activated genes were marked with green color.

Figure S2 (A&B) The cell proliferation of H446 lung cancer cells stably expressing the indicated overexpression or knockdown plasmids or a combination of plasmids was determined by the MTS assay. The error bar represents the SEM of triplicate experiments. The protein levels of SIRT2, or HRD1 or both were determined by western blotting using β-actin as loading control in H446 cells stably expressing control vector or indicated overexpression or knockdown plasmids. **(C-F)** A clonogenic assay was performed to measure the colony formation capacity of H446 lung cancer cells stably expressing the indicated overexpression plasmids (C&D) or knockdown plasmids (E&F). The quantitation of the colony number is shown (D&F). The results represent the mean±SEM of triplicate experiments. *$P<0.05$; **$P<0.01$. **(G-J)** Wound-healing assay was performed to measure the migration capacity of H446 lung cancer cells stably expressing the indicated plasmids or a combination of plasmids (G&I). The quantitation of the wound closure is shown (H&J). The results represent the mean ± SEM of triplicate experiments. *$P<0.05$; **$P<0.01$.
