# Supplementary figures and images for: E3 Ubiquitin Ligase HRD1 Promotes Lung Tumorigenesis by Promoting Sirtuin 2 Ubiquitination and Degradation
Source: Mol Cell Biol. 2020 Mar 16;40(7):e00257-19. doi: 10.1128/MCB.00257-19 (PMC7076256; doi:10.1128/MCB.00257-19)

Figure S1

A

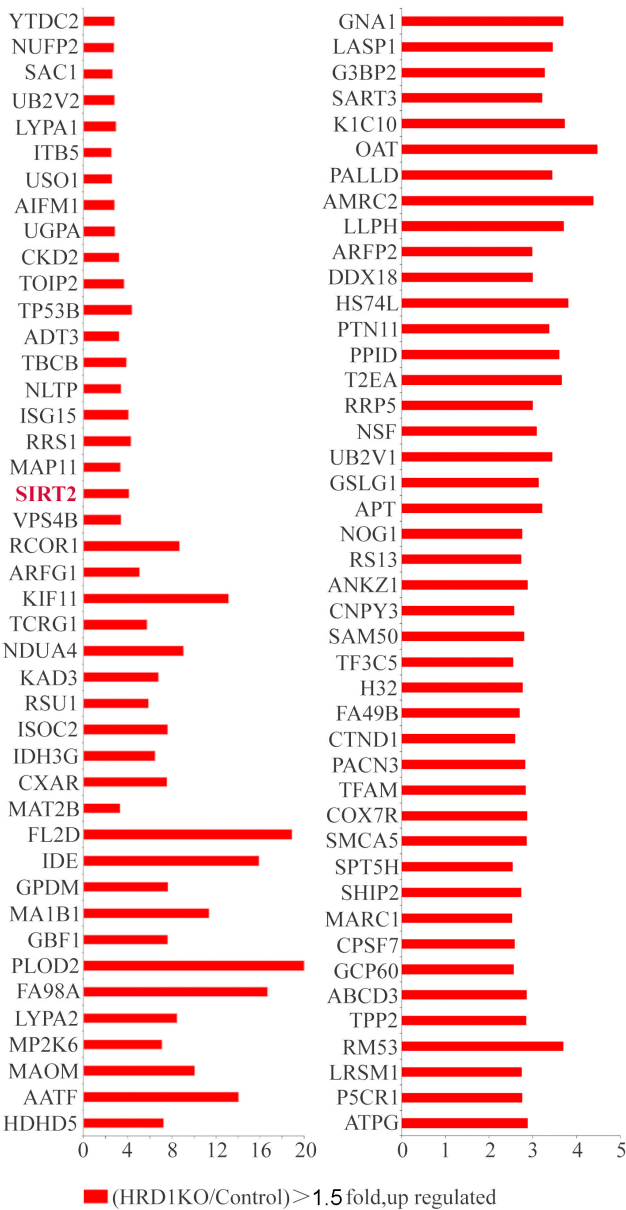

B

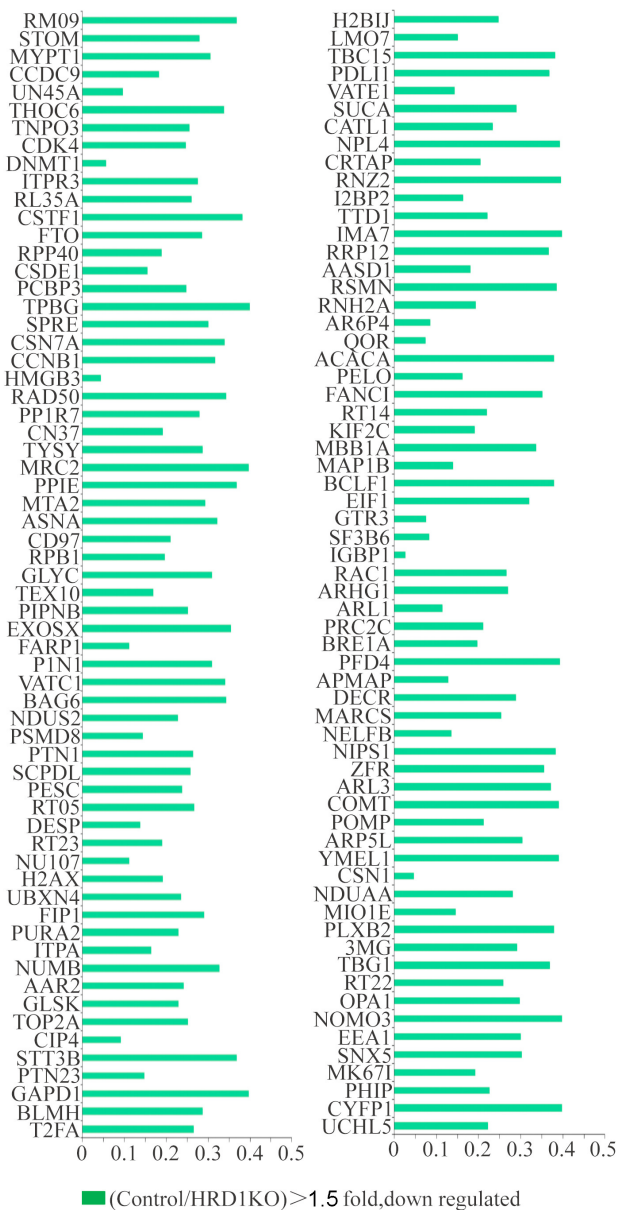

Supplement: Supplemental file 2 [file MCB.00257-19-s0002.pdf]

Figure S2

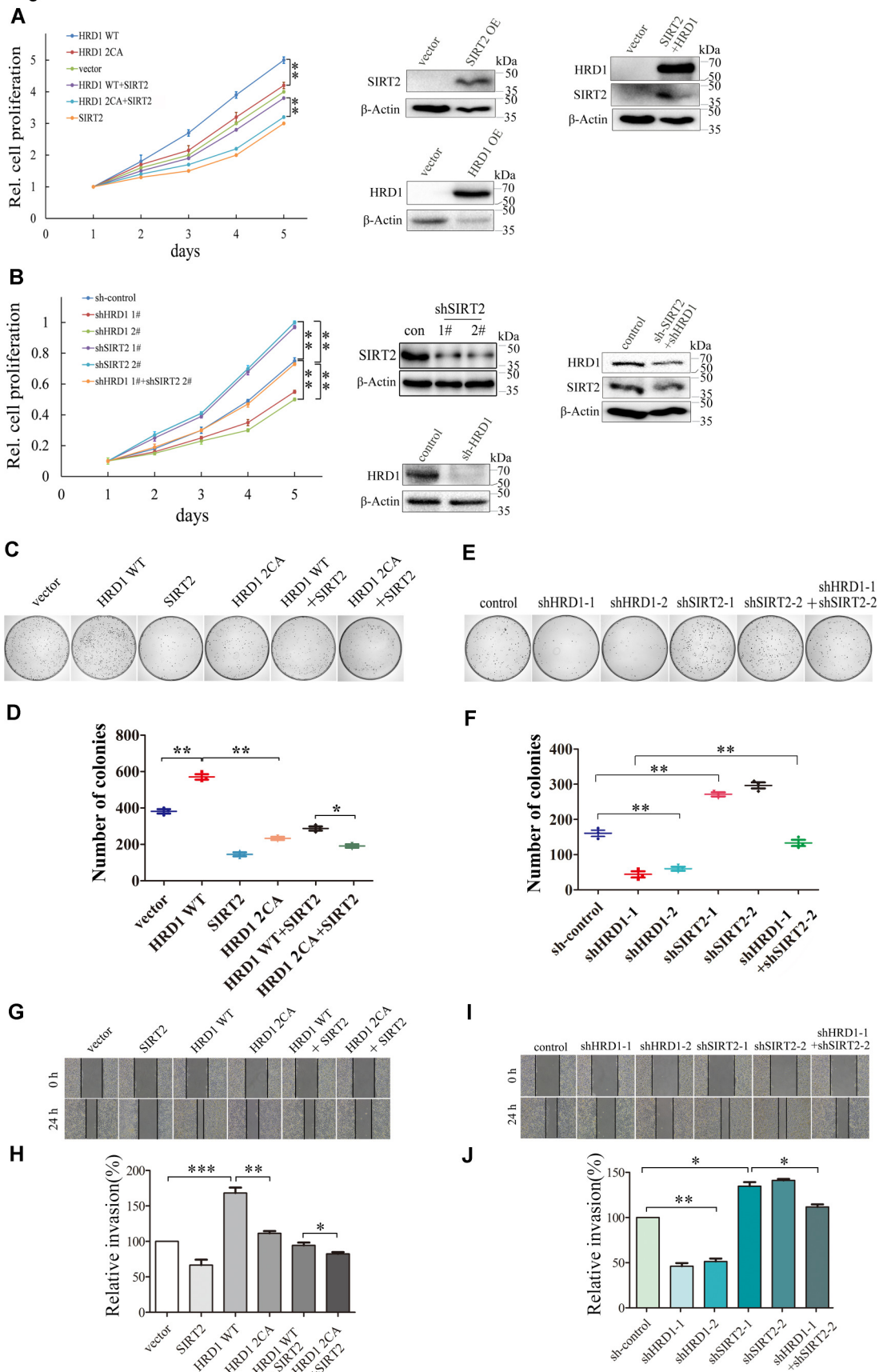

Supplement: Supplemental file 3 [file MCB.00257-19-s0003.pdf]
